# Structural Elucidation and Functional Characterization of the *Hyaloperonospora arabidopsidis* Effector Protein ATR13

**DOI:** 10.1371/journal.ppat.1002428

**Published:** 2011-12-15

**Authors:** Lauriebeth Leonelli, Jeffery Pelton, Allyn Schoeffler, Douglas Dahlbeck, James Berger, David E. Wemmer, Brian Staskawicz

**Affiliations:** 1 Department of Plant and Microbial Biology, University of California, Berkeley, California, United States of America; 2 QB3 Institute, University of California, Berkeley, California, United States of America; 3 Department of Molecular and Cell Biology, University of California, Berkeley, California, United States of America; 4 Department of Chemistry, University of California, Berkeley, California, United States of America; Virginia Polytechnic Institute and State University, United States of America

## Abstract

The oomycete *Hyaloperonospora arabidopsidis* (Hpa) is the causal agent of downy mildew on the model plant *Arabidopsis thaliana* and has been adapted as a model system to investigate pathogen virulence strategies and plant disease resistance mechanisms. Recognition of Hpa infection occurs when plant resistance proteins (R-genes) detect the presence or activity of pathogen-derived protein effectors delivered to the plant host. This study examines the Hpa effector ATR13 Emco5 and its recognition by RPP13-Nd, the cognate R-gene that triggers programmed cell death (HR) in the presence of recognized ATR13 variants. Herein, we use NMR to solve the backbone structure of ATR13 Emco5, revealing both a helical domain and a disordered internal loop. Additionally, we use site-directed and random mutagenesis to identify several amino acid residues involved in the recognition response conferred by RPP13-Nd. Using our structure as a scaffold, we map these residues to one of two surface-exposed patches of residues under diversifying selection. Exploring possible roles of the disordered region within the ATR13 structure, we perform domain swapping experiments and identify a peptide sequence involved in nucleolar localization. We conclude that ATR13 is a highly dynamic protein with no clear structural homologues that contains two surface-exposed patches of polymorphism, only one of which is involved in RPP13-Nd recognition specificity.

## Author Summary

Understanding how pathogenic microbes suppress host defenses and extract host nutrients is crucial to engineering methods to manage pathogen spread. By delivering an arsenal of proteins called effectors into the host, pathogens can overcome various counter measures taken by plants and animals to control pathogen proliferation. The key to deciphering how these pathogens manipulate their hosts is to determine the function of each effector and to evaluate its role in pathogen virulence. In the case of oomycetes, effectors share little sequence similarity to any known proteins; therefore, structural and functional predictions are difficult. By solving the structure of ATR13, we are able to contribute a protein structure to the PDB database and the scientific community at large. Our structure reveals the unique fold of our protein and illustrates how different evolutionary driving forces have shaped the surface topography of ATR13. Additionally, our structure allows us to identify a peptide sequence that plays a role in nucleolar transport, permitting us to inform nucleolar localization prediction programs about oomycete targeting sequences.

## Introduction

Oomycetes are a devastating class of filamentous eukaryotic pathogens that afflict plants and animals alike [1], [2]. Notorious for their role in the Irish Potato Famine and more recently for their decimation of the live oak species throughout California, oomycetes are highly pathogenic eukaryotic microbes that are difficult to control in the field—quickly overcoming chemical control methods and costing billions of dollars annually in crop losses [3], [4]. Despite the enormous impact of these pathogens, our knowledge of how they manipulate plant metabolism and overcome host defenses resulting in disease is still extremely limited. Many oomycetes are obligate biotrophs, making them difficult, if not impossible, to culture and are therefore genetically intractable. Phytopathogenic oomycetes like *Hyaloperonospora arabidopsidis* (Hpa) grow intercellularly, forming parasitic structures called haustoria that play a role in feeding and suppression of host defense systems. A cohort of pathogen proteins known as effectors are secreted across this haustorial membrane, a subset of which are further translocated across the plant plasma membrane by an unknown mechanism functional in both plants and animals [5], [6]. The role of most of these oomycete effectors in pathogen virulence has remained elusive, as many of their protein sequences lack similarity to proteins currently in the databases [7], [8].

The Hpa/*Arabidopsis* pathosystem is an ideal model for studying oomycete-host interactions. High levels of genetic diversity existing between naturally occurring populations of both Hpa and *A. thaliana*, along with genome sequence availability (www.arabidopsis.org, http://oomycetes.genomeprojectsolutions-databases.com/), allows for genetic exploration and dissection of each species. *ATR13* is an RxLR effector from the downy mildew oomycete *Hyaloperonospora arabidopsidis* (Hpa) that is recognized in *A. thaliana* in a race-specific manner by its cognate R-gene, RPP13 [9]. This class of proteins contains an RxLR motif that is implicated in host translocation. Both *ATR13* and *RPP13* are highly polymorphic genes, implying that the alleles have undergone diversifying selection at their respective loci [9], [10]. The maintenance of *ATR13* in all isolates of Hpa, together with the evidence of diversifying selection at this locus [11], implies that this effector confers a benefit to the invading oomycete. However, the function of *ATR13* has been difficult to extrapolate as no known proteins share sequence similarity to this effector.

Several effector molecules from other classes of pathogens have been structurally elucidated providing insight into their mode of action and virulence. The fungal effector AvrL567 from *Melampsora lini,* a flax rust, has similarity to ToxA [12], a protein involved in cell death induction from the necrotrophic wheat pathogen *Pyrenophora tritici-repentis* [13]. The NEP1-like effector, NLP_pya_, from the oomycete *Pythium aphanidermatum* has structural similarity to actinoporins, proteins derived from various marine invertebrates that form transmembrane pores facilitating membrane disintegration [14]. Additionally, crystal structures of bacterial effectors like AvrPto from *Pseudomonas syringae* in complex with their targets have provided a structural basis for the activation of plant immunity, showing how an effector interacts with its target and derepresses host defenses [15]. Recently, there has been a surge of structural information becoming available pertaining to RXLR effectors. The NMR structure of the *Phytophthora capsici* RXLR effector Avr3a4, a close homolog to the *P. infestans* Avr3a that inhibits CMPG1 function *in planta* [16], revealed a positive surface patch involved in binding phosphatidylinositol monophosphates (PIPs)—compounds essential for Avr3a accumulation and therefore function [17]. Chou et al. solved the crystal structure of ATR1, an Hpa RXLR effector that adopts a two-domain structure comprised of 13 α-helices. Mapping by sequence conservation among ATR1 alleles revealed that polymorphic residues specifying RPP1 recognition were distributed in clusters along the surface of the protein [18]. Interestingly, Boutemy et al. have used structural biology and bioinformatics to show that Avr3a11 from *P. capsici* and PexRD2 from *P. infestans* share a conserved α-helical fold (termed the WY domain) along with a predicted 44% of all annotated *Phytophthora* RXLR effectors [19] and the ATR1 protein described by Chou et al [18].

To obtain more information on the virulence function and recognition domains of ATR13, we used Nuclear Magnetic Resonance (NMR) to solve its backbone structure. Further, we generate loss-of-recognition and gain-of-recognition mutants through both site-directed and random mutagenesis and map these mutations onto the structure to identify regions important in RPP13 recognition. Additionally, we describe a region of ATR13 required for nucleolar localization but show that ATR13 subcellular localization has no effect on recognition by RPP13. Mutational effects of ATR13 Emco5 (recognized by RPP13) and ATR13 Emoy2 (unrecognized by RPP13) are assayed using the *Agrobacterium tumefaciens/ Nicotiana benthamiana* surrogate system [20], where ATR13 recognition by RPP13, in this case the Niederzenz allele (RPP13-Nd), results in the hypersensitive response (HR), a plant-specific form of programmed cell death purported to limit pathogen spread.

## Results

### ATR13 protein expression and deletion analysis

**Figure 1:**
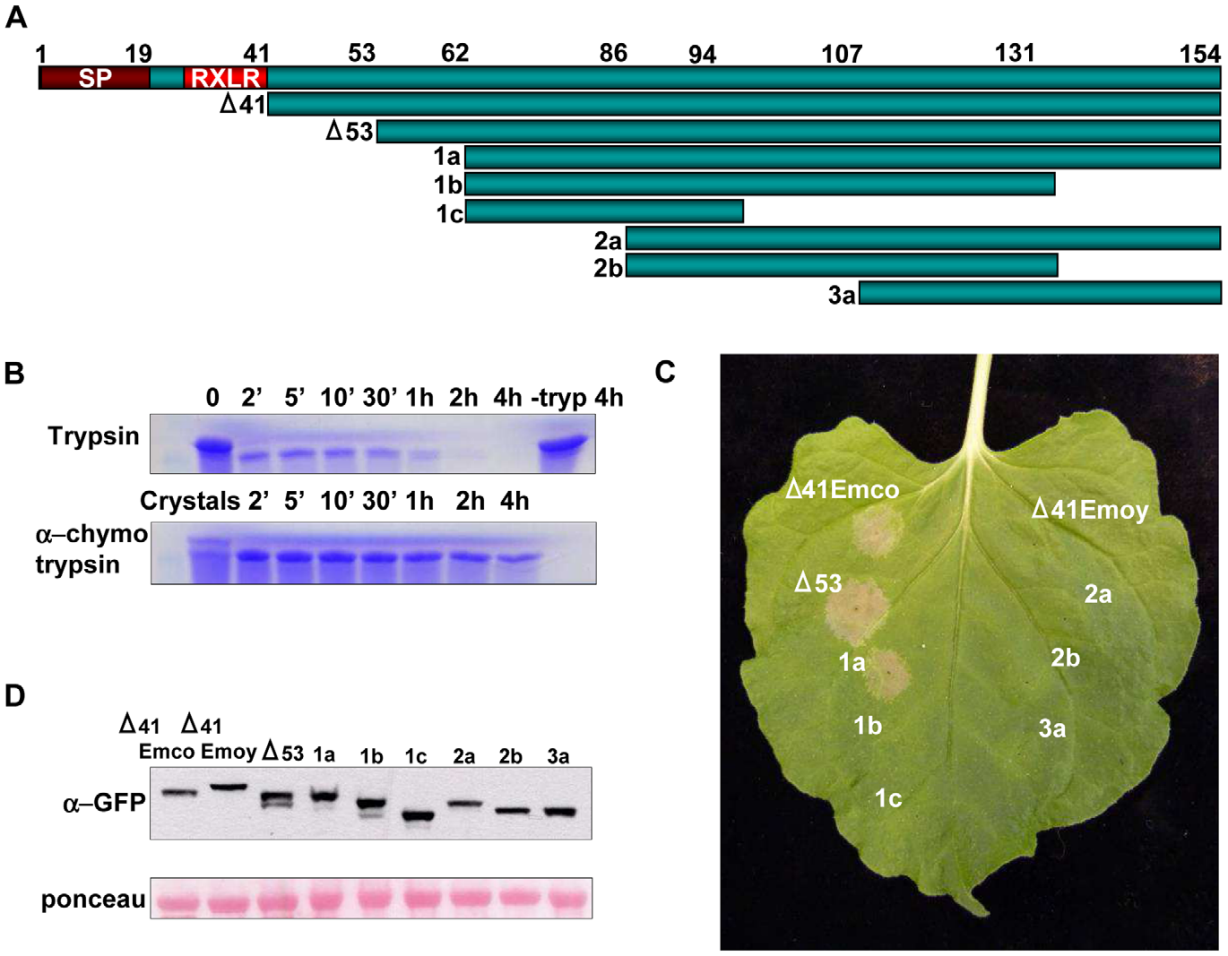
Determining Active Truncations of ATR13 Emco5. A. Schematic diagram of ATR13 Emco5 truncations in pEarleygate101 assayed for HR-inducing activity. Features include a signal peptide involved in effector secretion and an RxLR motif implicated in host-translocation. **B.** Coomassie-stained SDS-PAGE gel showing limited proteolysis of purified Δ41ATR13 Emco5 using trypsin and α-chymotrypsin. Twenty crystals were harvested, dissolved, and run with the α-chymotrypsin samples. **C.** Transgenic Nicotiana benthamiana containing RPP13-Nd recognizing various transiently expressed ATR13 Emco5 truncations. **D.** Western blots probed with GFP antibody and loading-control ponceau stain of protein extracted from N. benthamiana transiently expressing ATR13 Emco5 truncations.

To obtain soluble protein for structural studies, we expressed truncations of three different alleles of ATR13: Emco5, Maks9, and Emoy2, lacking the secretion peptide and RxLR translocation domain (Δ41 truncations) (Figure 1A). Of the three alleles, ATR13 Emco5 produced the most soluble protein and was therefore selected for generation of crystals for structural determination. Efforts to crystallize ATR13 were successful (Figure S1A–C); however, we were unable to determine experimental phases. From our crystallographic efforts, we noticed that crystals required a minimum of two months to form, leading us to believe that some kind of natural proteolysis was taking place prior to crystallization. To address this, we performed limited proteolysis [21] using both trypsin and chymotrypsin on Δ41 ATR13 Emco5 samples to discover a more stable truncated version of the protein (Figure 1B). Additionally, we determined that the protein in the crystals existed in two forms: the original Δ41 version and a cleaved version we identified as Δ53 ATR13 by mass spectrometry.

To verify the biological relevance of our Δ53 ATR13 Emco5 truncation, we transiently expressed this truncation in *N. benthamiana* containing the RPP13-Nd transgene, and demonstrated its ability to trigger a hypersensitive response (HR). Furthermore, to determine the minimal region necessary for RPP13-Nd recognition, truncations from both the N-terminus and C-terminus of ATR13 Emco5 were expressed transiently in *N. benthamiana* via *Agrobacterium* inoculations. While RPP13-Nd was able to recognize ATR13 Emco5 N-terminal truncations up to 62 amino acids, once 86 amino acids were removed, RPP13-Nd recognition was compromised (Figure 1C). All C-terminal deletions resulted in compromised recognition, despite intact protein expression (Figure 1D).

### ATR13 structures by NMR

**Figure 2:**
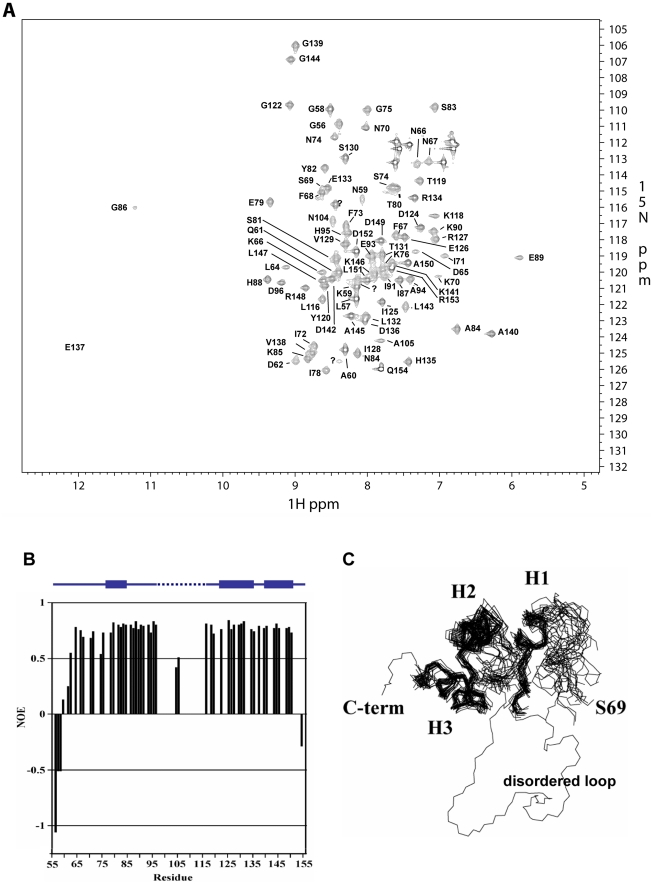
NMR analysis of ATR13 Emco5. A. 15 N-HSQC of Δ53 ATR13 Emco5. Assignments are denoted by one-letter amino acid code and sequence number. With the exception of N104 and A105, signals were not observed for the flexible region (L97 through Y115), likely due to dynamics on an intermediate time scale. **B.** Heteronuclear NOE diagram of ATR13 Emco5 showing dynamics of ATR13 Emco5 residues. Flexible residues have values below 0.5. Secondary structure is shown above the NOE panel; blue rectangles denote helices, line denotes coiled coil regions, and dashed line indicates disordered residues. **C.** Overlay of the twenty lowest energy structures forming a consensus at the C-terminus and part of the N-terminal region. The two regions are connected by the disordered region depicted here by a representative loop from one of the twenty structures. Alpha helices are denoted as H1: residues 77–85, H2: residues 122–135, and H3: residues 140–150.

**Figure 3:**
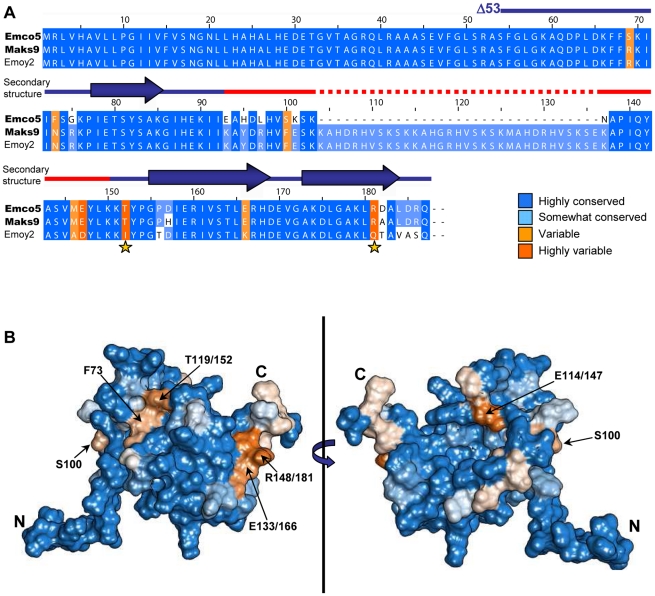
Naturally occurring polymorphisms of ATR13. A. A clustalX alignment of ATR13 variants colored by percent conservation calculated from 15 isolates in Jalview; three are shown. ATR13 alleles recognized by RPP13Nd are shown to the left in bold. Residues previously implicated in recognition are marked by yellow stars. Secondary structure is represented above corresponding residues; lines denote coiled regions whereas arrows denote alpha helices. The red section marked on the secondary structure indicates disordered residues. **B.** Naturally occurring polymorphisms mapped onto a representative ATR13 structure rendered in Chimera. Polymorphic residues are shown in orange while conserved residues are shown in blue.

After attempts to solve the structure using crystallography stalled, we turned to nuclear magnetic resonance spectrometry (NMR) to solve the structure of ATR13. Our first ^1^H-^15^N heteronuclear single quantum coherence (HSQC) spectrum provided insight as to why crystallography was unsuccessful; only three-fourths of the expected signals were observed, suggesting that the remainder of the protein was not well ordered (Figure 2A). Worried that this region of internal disorder was due to several missing direct repeats in the Emco5 allele relative to other alleles of ATR13 (Figure 3A), we purified ATR13 Maks9 and collected its ^1^H-^15^N HSQC spectrum. The ^1^H-^15^N HSQC spectrum of the Maks9 allele contained approximately the same number of peaks as the Emco5 allele, suggesting that the insertion present in the longer alleles does not stabilize the disordered loop. Overlays of the Maks9 and Emco5 spectra also reveal significant overlap between peaks present in both samples, indicating that additional missing peaks in the Maks9 spectrum are most likely part of the direct repeat region not found in Emco5.

Backbone amide proton assignments were obtained using standard 3D triple resonance heteronuclear experiments for 81 residues including those in segments G56 through D96, N104 and A105, and L116 through Q154 with the exception of I92. Only one sharp peak and two broad peaks remain unassigned in the HSQC spectrum, indicating that approximately 16 of the 95 expected HN signals are missing. By process of elimination, we conclude that these missing signals correspond to residues in segment 97 – 115, as all other residues have backbone amide proton assignments. Of the residues with assigned HN resonances, approximately 88% of the side chain signals were also assigned. The chemical shift assignments were combined with NOE, J_HNHα_ scalar couplings, hydrogen exchange measurements, and residual dipolar couplings to construct a structure of ATR13. A summary of the NMR-derived restraints and structural statistics are presented in Table 1. The well-ordered region of the structure (76–88 and 120–150) is defined by 14 restraints per residue. These data yielded a well-defined backbone fold, but the sidechains are less well defined. Restraint violations for ATR13 are good, as are most of the structural quality factors (Table 1). The relatively high value obtained from Verify3D [22] is due to the ill-defined state of the loop region (residues E89 – Y115) (see below). The portion of ATR13 elucidated by NMR consists of a central helix (residues 122–135) that packs against a short helix and turn (P77-H88) on one side, and a long C-terminal helix (residues A140 – A150) on the other (Figure 2C).

**Table 1:** NMR parameters, restraints, and statistics of the ATR13 structure.

ATR13 Structural Restraints and Statistics^A^			
NOE			448
	Intra	i = j	155
	Sequential	|i-j| = 1	142
	Medium	|i-j| < 5	80
	Long	|i-j| > 5	71
Dihedral			43
HN RDC			27
Hydrogen Bond^B^			28
**Restraint RMSDs^C^**			
NOE (Å)			0.025 +/- 0.004
Phi Dihedral (deg)			0.2 +/- 0.2
RDC (Hz), Qrdc			0.8 +/- 0.2 , 7.4%
Hydrogen Bond (Å)			0.029 +/- 0.006
**Coordinate RMSDs^D^**			
Backbone			0.8 Å
Heavy atom			1.5 Å
**Close contacts(per structure)^E^^,^^F^**			0.2
**Ramachandran analysis^D^^,^^E^**			
Most favored region (%)			83.2
Additionally allowed (%)			14.9
Generously allowed (%)			1.6
Disallowed (%)			0.3
**Structure Quality Factors^E^^,^^G^**			
Verify3D			−5.14
Prosall			−1.99
Procheck	(phi-psi)		−2.28
Procheck	(all)		−4.7
MolProbity			−1.36

A. Parameters are for the 20 best of 200 structures.

B. Two restraints per hydrogen bond for a total of 14 hydrogen bonds.

C. For NOEs, on average, there was one NOE violation greater than 0.2 Å per structure, with the maximum violation equal to 0.5 Å. For phi dihedral restraints, on average, there were 0.4 violations greater than one degree, with the maximum violation of four degrees. For residual dipolar couplings, on average, there were 0.8 violations greater than 1.5 Hz, with the maximum violation equal to 3.4 Hz. For hydrogen bonds, on average, there were 0.4 violations greater than 0.1 Å, and all were less than 0.2 Å.

D. Includes residues 76–88, and 120–150.

E. Determined with the Protein Structure Validation Suite (PSVS) version 1.4.

F. Close contacts are defined as within 1.6 Å for H atoms, 2.2 Å for heavy atoms.

G. With respect to mean and standard deviation for a set of 252 X-ray structures < 500 residues, of resolution < =  1.80 Å, R-factor < =  0.25 and R-free < =  0.28.

The N-terminal residues prior to the first helix (G54-P77) are not particularly well defined by the Nuclear Overhauser Effect (NOE) data. As an alternative, the steady-state ^1^H-^15^N NOE enhancement provides a qualitative measure of dynamics [23]. Rigid HN bonds typically have NOE enhancements of approximately 0.8. As the sub-nanosecond dynamics increase, the NOE enhancement decreases, and can even become negative. Heteronuclear NOE values for residues G56 through D62 increase slowly from −1.0 to 0.5, characteristic of a flexible N-terminus. However, there is some evidence that residues L64 through K76 are more ordered than could be defined. For example, the program TALOS [24], which compares measured CA, CB, CO, and HA chemical shifts to those from a database of known structures, predicts that the phi / psi angles for residues L64-K70 adopt a helical conformation, while in the later portions of the segment (S69-K76) several weak dNN NOEs and small ^3^J_HNHA_ couplings (∼5 Hz) suggest a turn or helical structure. These data give rise to the hint of structure for S69 through K76 (Figure 2B, C). Residues L64 through K76 also have fairly high heteronuclear NOE values (∼0.7) which supports the premise that there is some order within this region that is not defined by the NMR data.

The most outstanding feature of the structure is an ill-defined loop that extends from E89 to Y115. In the segment 97 – 115, only N104 and A105 are assigned. N104 and A105 show reduced ^1^H-^15^N heteronuclear NOE values (∼0.5), also suggesting that at least a portion of the segment is flexible (Figure 2B). In attempts to solve the crystal structure, density for the single selenomethionine (M113) located within the loop region was displaced from the remaining protein density, which is consistent with disorder in this region. As stated earlier, approximately 16 amide signals were missing from the ^1^H-^15^N HSQC spectrum, most of which correspond to residues within this region. The absence of peaks suggests that this loop has flexibility on an intermediate time scale under the conditions studied. Structural homology searches using the Dali server (http://ekhidna.biocenter.helsinki.fi/dali_server/start) yielded few candidate proteins with very weak structural similarity (Figure S2) indicating that ATR13 possesses a fold that has not yet been described in the PDB databank.

### Polymorphic residue clustering on the surface of ATR13

Using sixteen alleles of ATR13 (Figure S3), we generated an alignment in Jalview [25] and determined percent conservation of residue identity across ATR13 alleles using BLOSUM [26]. Conservation scores were mapped onto a single representative low-energy model of the ATR13 Emco5 structure (used throughout) with the Chimera software package [27], displaying highly polymorphic residues in shades of orange and conserved residues in shades of blue (Figure 3). This model shows two discreet pockets of polymorphism on the surface of the ATR13 protein. Additional low energy models of ATR13 Emco5 displaying percent conservation show similar polymorphic patches (Figure S4).

### Loss of Recognition (LOR) by Site Directed Mutagenesis (SDM) of ATR13 Emco5

**Figure 4:**
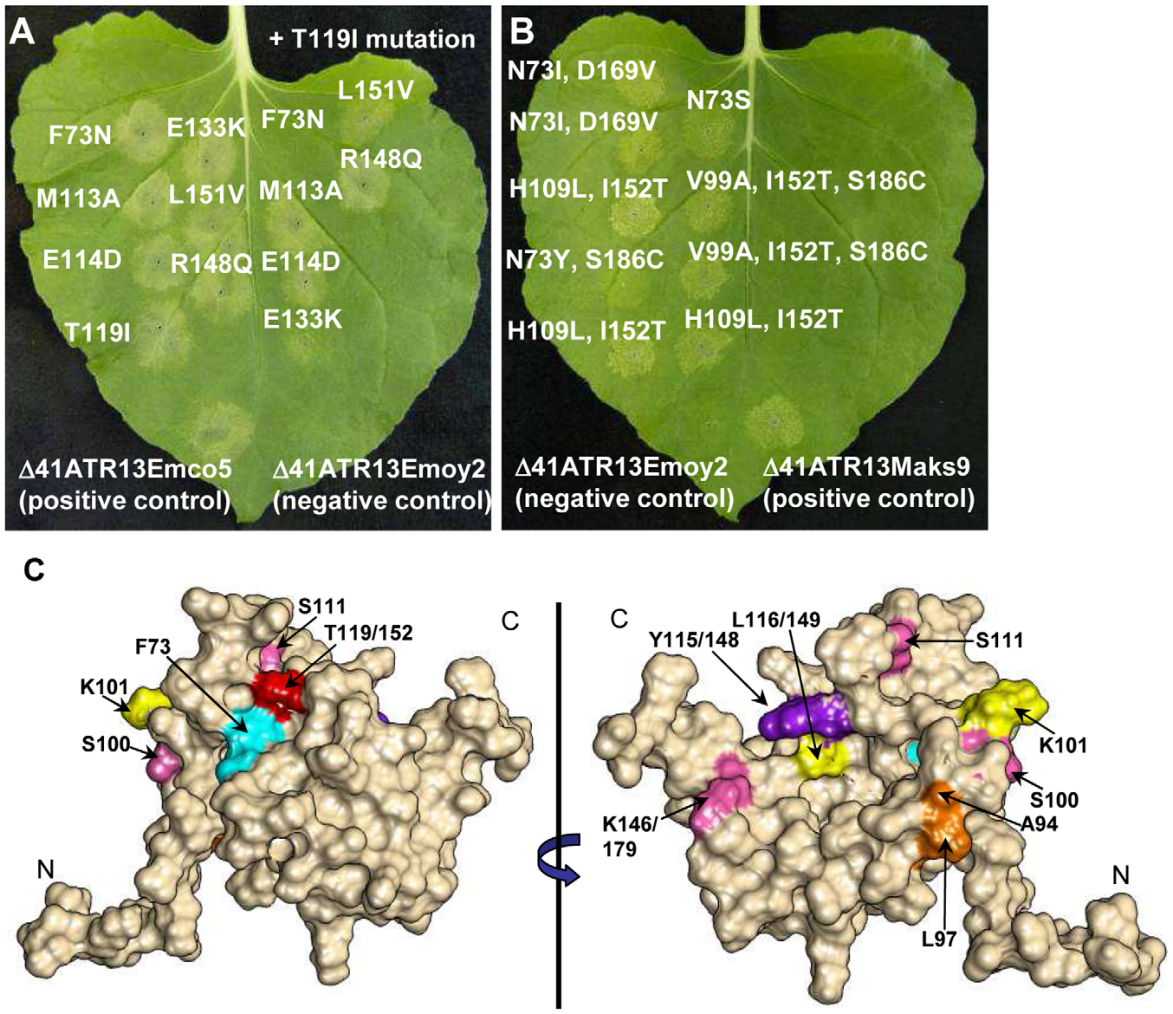
Site-directed loss-of-recognition (LOR) and random gain-of-recognition (GOR) mutagenesis of ATR13 scored for HR in RPP13 transgenic N. benthamiana plants. **A.** Site-directed mutation of residues from ATR13 in the Emco5 allele to those found in the unrecognized Emoy2 allele producing LOR by RPP13Nd. Inoculations on the right of the leaf are double mutants containing the T119I amino acid change. Note that residue numbers correspond to amino acid positions in Emco5. **B.** Residues mutated in the Emoy2 allele of ATR13 producing GOR by RPP13Nd. The numbering of these residues corresponds to amino acid positions in Emoy2, for example T119 Emco aligns structurally with I152 Emoy. **C.** F/N73 (teal) and T(I119)152 (red) residues mapped onto the ATR13 structure. Additionally, mutants generated from random loss-of-recognition mutagenesis that maintained ATR13 at the wildtype level are shown. Colors indicate mutations that occurred together to compromise RPP13 recognition. Where different, residue positions are listed relative to Emco5 position first and Emoy2 position second.

Exploiting natural variation occurring between recognized and unrecognized alleles of ATR13 , Emco5 and Emoy2 respectively, we singly or doubly mutated polymorphic residues possessing vastly different chemical properties between alleles, or residues previously implicated in recognition (Figure 3A) [11]. Using the surrogate *Agrobacterium/N.benthamiana* system [20], we demonstrated that single amino acid changes of these residues have little to no effect on recognition of ATR13 Emco5 by RPP13Nd. However, in several cases, double mutations reduced the intensity of the hypersensitive response (E133/166K, T119/152I and T119/152I, R148/181Q) or eliminated it, as is the case with F73N, T119/152I (Figure 4A). When mapped onto the structure, F73 and T119/152 appear to be surface-exposed and in close proximity (Figure 4C), implicating this specific region in avirulence determination.

### Gain of Recognition (GOR) by random mutagenesis of ATR13 Emoy2

As a complement to the loss-of-recognition screen, we performed gain-of-recognition random mutagenesis on ATR13 Emoy2. After screening 800 mutant ATR13 Emoy2 alleles for altered recognition by RPP13-Nd on *N. benthamiana*, we identified nine clones that possessed an intermediate recognition phenotype (Figure 4B). All nine of these mutants had either the I119/152T, or N73Y/S/I substitutions, lending support to the theory that these two residues are critical for RPP13-Nd mediated HR. In addition, like unrecognized alleles, the Maks9 variant of ATR13 has an asparagine at residue 73, however it is recognized by RPP13-Nd. In this allele, when N73 is substituted with a phenylalanine like that found in Emco5 and most recognized alleles, the resistance response by RPP13-Nd is more robust than that generated against wildtype Maks9 (data not shown) again implicating this residue position as crucial for full RPP13-Nd recognition.

### Loss of Recognition (LOR) by random mutagenesis of ATR13 Emco5

To more thoroughly explore the avirulence role of ATR13 in conjunction with RPP13-Nd, we performed random mutagenesis to identify additional amino acids that play a role in ATR13 recognition. Of 1,200 colonies screened, 95 clones showed a loss-of-recognition phenotype. When sequenced, 50 of these clones had either frame shift mutations or early stop codons, while the remaining 45 had either single, double, or triple mutations (Figure S5C). We also sequenced 95 mutant clones showing intact HR signaling. These retention-of-recognition (ROR) mutants were used to eliminate background mutations that did not alter recognition (Table S1). When inoculated onto *N. benthamiana,* the 45 LOR mutants display varied timing and intensity of hypersensitive response, as well as a range of mutant protein stabilities relative to wildtype levels (Figure S5A, B). Fourteen mutant alleles of ATR13 Emco5 appear to accumulate amounts of protein equaling or in excess of the wildtype level (Figure S5C), and when analyzed in the context of the structure, these residues are nested within its core rather than surface-exposed (Figure 4C), suggesting that the overall fold of the protein is altered rather than the interaction surface. Interestingly, most of the altered residues occur in regions that are conserved among natural ATR13 variants. When we mutate one of these conserved residues, Y115/148N, from another recognized allele of ATR13, Maks9, we again abolished recognition by RPP13Nd, showing that the altered phenotype is not specific to the mutant ATR13 Emco5 (data not shown).

### ATR13 localization and identification of a Nucleolar Localization Sequence (NoLS)

**Figure 5:**
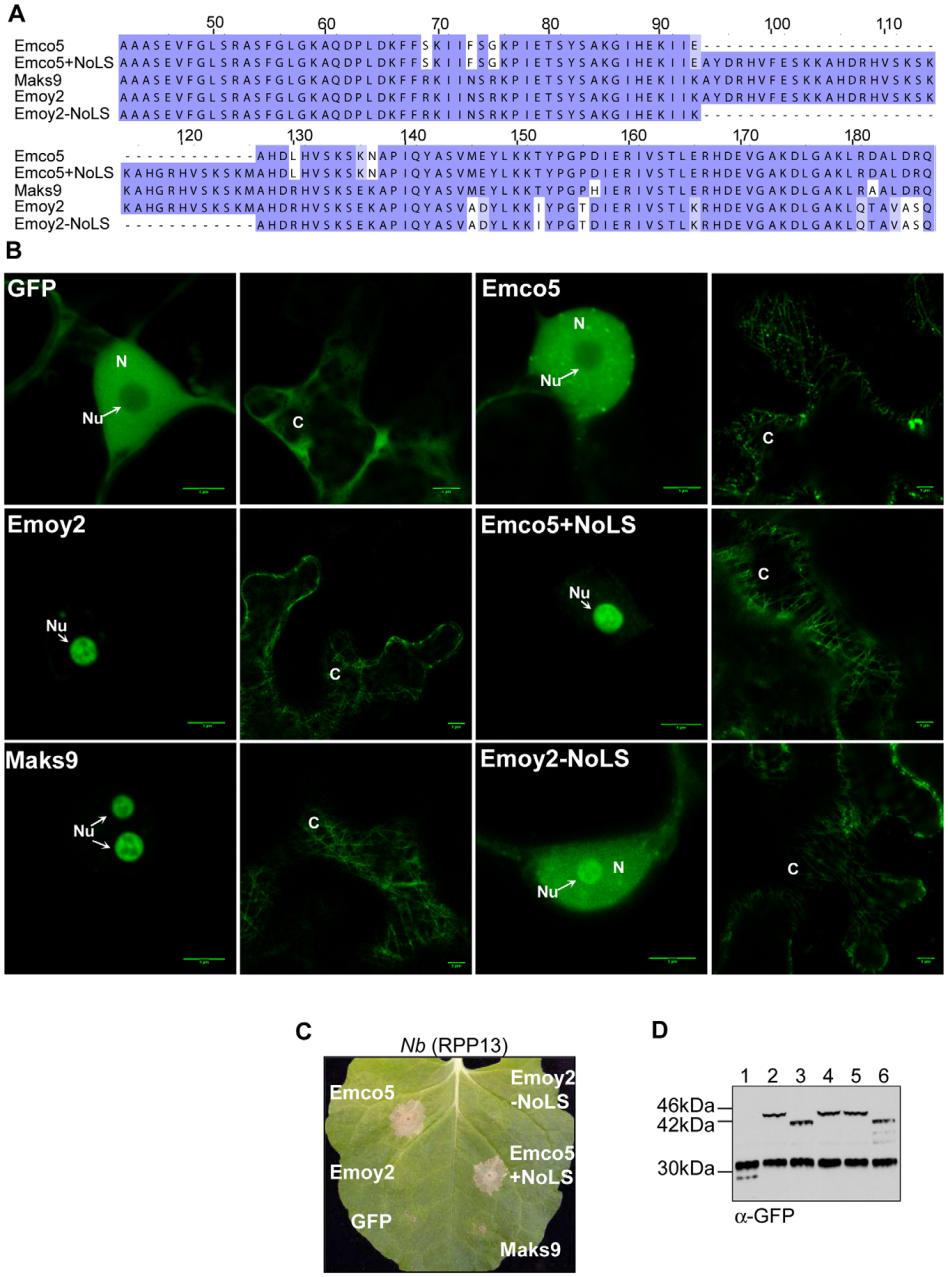
Nucleolar targeting signal of ATR13. A. An alignment of various ATR13 chimeras showing the naturally occurring insertion present in Maks9 and Emoy2 alleles of ATR13, the insertion added to the Emco5 allele, and the deletion from the Emoy2 allele. **B.** Localization of GFP-fused ATR13 chimeras expressed transiently in N. benthamiana. The left panels are focused on nuclei (N) and nucleoli (Nu), whereas panels to the right are images of associated cytoplasm (C). Scale bars are 5 um. **C.** Expression of these constructs in N. benthamiana containing RPP13Nd showing intact recognition patterns despite altered localization. **D.** Western blot of various ATR13 alleles and chimeras probed with α-GFP showing comparable expression levels in N. benthamiana. Lanes are labeled as following: 1. 35S-GFP, 2. 35S-Emoy2:GFP, 3. 35S-Emco5:GFP, 4. 35S-Maks9:GFP, 5. 35S-Emco5+ NoLS:GFP, 6. 35S-Emoy2-NoLS:GFP.

The disordered residues in the Emco5 allele of ATR13 flank an insertion present in other alleles, including Maks9 and Emoy2, both of which we have observed in the nucleolus. To assess whether this 33 amino acid insertion was responsible for nucleolar targeting, we embedded this sequence at the analogous position in the Emco5 allele (Figure 5A), usually excluded from the nucleolus and present in both nucleus and cytoplasm. We show that the addition of this 33 amino acid insertion results in a dramatic change in localization of the Emco5 allele—the chimeric form of ATR13 Emco5 becomes highly enriched in the nucleolus (Figure 5B). To check if the deletion of this insertion in the Emoy2 allele abrogated nucleolar localization, we removed these 33 amino acids and determined that while still present to a lesser degree in the nucleolus, Emoy2 was now present throughout the nucleus, similar to the wildtype Emco5 localization pattern. Despite the change in localization of these two alleles, RPP13-Nd recognition remained unaltered; Emco5+NoLS is still recognized and triggers HR, whereas Emoy2-NoLS remains unrecognized (Figure 5C).

It is also worth noting that in addition to nuclear or nucleolar localization, the cytoplasm appears to undergo dramatic changes when any of the alleles of ATR13 (Emco5, Maks9, and Emoy2) are expressed *in planta.* Relative to GFP, ATR13 appears to localize to distinct cytoplasmic strands, as well as to punctate bodies associated with these cytoplasmic strands and throughout the cytoplasm. The cytoplasmic patterning associated with ATR13 Emco5 is quite dramatic, displaying an abundance of punctate spots throughout the cytoplasm.

## Discussion

The structure of ATR13 from Emco5 was determined to moderate resolution using NOE, ^1^J_HNHA_ scalar coupling, hydrogen exchange, and residual dipolar coupling data. The presence of significant disordered regions, somewhat poor magnetization transfer, and in some cases peak overlap, hampered our efforts to obtain a structure of higher resolution. Nevertheless, the structure was of sufficient quality to permit comparison with other proteins in the protein data bank. Despite very weak resemblance to several proteins including GTP-binding nuclear RAN, Beta-1 subunit importin, and a serine/threonine phosphatase 2A, the global fold of ATR13 from Emco5 appears to have no obvious homology to known proteins in the PDB database. In contrast to other RXLR proteins, ATR13 does not possess the core α-helical fold that is conserved in ATR1, Avr3a11, and PexRD2 [18] [19].

ATR13 is a highly polymorphic protein, yet only a small subset of the polymorphic residues appear to be involved in RPP13Nd-mediated recognition. As is the case with ATR1, polymorphic residues of ATR13 appear as clusters across the surface of the protein. We have shown two major surface-exposed patches on the ATR13 structure that are highly polymorphic, yet only one of these regions appears relevant to RPP13Nd recognition. Previous studies implicate E(114)147, T(119)152, and R(148)181 as being essential for full RPP13Nd recognition of the Wela3 and Maks9 alleles of ATR13 [11]. Here we show that ATR13 Emco5 recognition is mediated specifically by F/N73 and T/I(119)152 substitutions, as determined in both loss and gain-of-recognition mutagenesis screens. When mapped onto the structure of ATR13 Emco5, these two residues are in close proximity and are solvent-exposed, suggesting a surface-exposed patch that is required for RPP13-Nd recognition. Hall et al. have shown that several *Arabidopsis* accessions contain R-genes other than RPP13 that function in ATR13 recognition [28]. These other R-genes, as well as other functional alleles of RPP13, may serve as a driving force behind other polymorphic patches in ATR13. It will be interesting to see if recognition conferred by these R-genes is affected when residues in either surface-exposed patch of ATR13 are mutated, or if the same LOR and GOR mutants identified in this study maintain their phenotype in the context of this other R-gene. Many of the residue changes uncovered during the LOR random mutagenesis screen occurred in residues that are conserved in both recognized and unrecognized alleles of ATR13. There are distinct differences in timing and intensity of hypersensitive response, implicating these residues in proper folding or stability. However, several mutants appear to accumulate protein to the wildtype level, and at least one of these mutations, Y(115)148N, also alters RPP13-Nd recognition of ATR13 Maks9. Notably, in several of the NMR models this tyrosine is proximal to N73 or T(119)152, suggesting it may directly affect the orientation and accessibility of these residues.

The disordered loop is one of the most interesting features of the ATR13 structure. This portion of ATR13 Emco5 flanks one of four 11 amino acid direct repeats found in other alleles of ATR13 [11]. These other alleles are shown to localize to the nucleolus when expressed *in planta*, whereas ATR13 Emco5 does not. When these three missing direct repeats are added to the ATR13 Emco5 allele, the chimera relocalizes to the plant nucleolus, suggesting that this region is involved in nucleolar localization. Nucleolar localization is difficult to predict, as little data is currently available regarding how proteins are targeted to the nucleolus [29], however several hallmarks of nucleolar localization signals (NoLS) include surface exposed coiled coil domains containing an abundance of lysines or arginines [30]. In the 33 amino acid stretch that defines the nucleolar targeting sequence, lysines and arginines account for nearly one-third of residue content. Additionally, regions of disorder often require one or several ligands for stabilization [30]. This region of ATR13 could potentially bind rRNA, rDNA, or a protein involved in nucleolar trafficking. Thus far, this is one of the only described examples of an oomycete protein localizing to the plant nucleolus. Moreover, it is functional in the host rather than in the originating pathogen, suggesting a signaling hierarchy; secretion and translocation across the host plasma membrane occurring prior to nucleolar targeting.

The nucleolus is best known for its role in ribosome biosynthesis, yet it is also essential for regulating the cell cycle and the cellular response to stress. In humans a mere 30% of known nucleolar proteins play a role in ribosome biosynthesis, whereas the remaining 70% play various roles in cell maintenance, apoptosis, DNA replication and repair, cell cycle control, and stress signaling [31]. In plants, the nucleolus has been shown to be a target of several pathogen classes, including a groundnut rosette virus that recruits RNA processing machinery to produce viral RNP (ribonucleoprotein) particles needed for systemic infection [32]. For the Picorna-like Potato virus A, the nucleolar localization of one of its proteins, Nla, is required for completion of its infection cycle on *Nicotiana* [33]. *Globodera pallida*, a potato cyst nematode, has also been shown to target the nucleolus during various life stages presumably to suppress host defense [34]. Interestingly, Gilroy et al. (2011) show that the host protein CMPG-1, an E3 ligase involved in resistance signaling, accumulates in the nucleolus when the *P. infestans* effector Avr3a is transiently expressed in *N. benthamiana* [35]. With the varied roles the nucleolus plays in directing cellular activities, it seems an attractive target for an intercellular obligate biotroph requiring compromised host defense and a steady supply of nutrients. In light of our findings, examining the role of the nucleolus in oomycete pathogenesis is an area that requires further exploration. Knowing that several alleles of ATR13 are localized to the nucleolus, a structure necessary for a variety of cellular processes including the cellular stress response, we might look more closely at its role during pathogenesis and determine if known nucleolar controlled stress responses are altered upon challenge with pathogen-delivered ATR13.

In addition to its nuclear and nucleolar localization, ATR13 appears to localize to the cytoplasmic scaffolding and to discrete punctate spots along these strands. As an obligate biotroph, nutrient acquisition is one of the key factors influencing survival and success of the invading pathogen. To that end, hijacking cellular transport machinery would be an effective strategy for funneling nutrients from plant host to obligate pathogen. The various cellular localizations of ATR13 suggest it may possess multiple roles in pathogenesis, much like the EspF effector from *Escherichia coli* which has been shown to target the mitochondria, nucleolus, and cytoplasm of infected mammalian cells [36].

In this study we solve the structure of ATR13, a structurally flexible and highly polymorphic effector protein from Hpa. We infer that its maintenance in Hpa, in spite of the drive to evade host recognition by RPP13, illustrates its importance in pathogen virulence—especially in the context of Hpa's abbreviated effector repertoire [37]. We identify two ATR13 residues essential for robust HR in the presence of RPP13Nd. We map these residues onto our structure and show that they localize to a single solvent-exposed patch which corresponds to an area under high diversifying selection. Lastly, we show that the highly flexible internal loop we identified based on our NMR data plays a role in nucleolar localization and can be added to a non-nucleolar protein to redirect that protein to the nucleolus. 

## Materials and Methods

### Native protein expression

pET-DUET1 constructs were transformed into chemically competent Rosetta(DE3)pLysS *E. coli* (Novagen), and selected on LA plates containing 50 ug/ml carbenicillin. Single colonies were used for overnight starter cultures and diluted to an OD of 0.1 in LB +carb the following morning. These cultures were incubated at 37°C and agitated at 250 rpm until reaching an OD of 0.55. Induction was initiated by the addition of IPTG to a final concentration of 500 uM. Cultures were induced for 16 h at 28°C and 250 rpm and cells were harvested by centrifugation at 3,000 rpm. Cells were resuspended in a small volume of buffer A (20 mM phosphate buffer pH 7.2, 20 mM imidazole, 0.5 M NaCl, 10% glycerol), snap frozen in liquid nitrogen, and stored at −80°C.

### Labeled protein expression

Overnight starter cultures were prepared as described above, spun down at 3,000 rpm for 15 minutes and washed once in M9 minimal media. For NMR experiments, uniformly ^15^N-labeled and uniformly ^15^N/^13^C-labeled ATR13 were expressed in *E. coli* using M9 minimal medium containing either ^15^N-labeled ammonium chloride, or ^15^N-labeled ammonium chloride and ^13^C-labeled glucose (Cambridge Isotopes Laboratories). A 10% fractionally ^13^C-labeled sample was prepared by growing the bacteria in medium containing 10% ^13^C-labeled glucose. Protein yields ranged from 20 to 25 mg per liter.

### Protein purification

Frozen cell suspensions were thawed and incubated with 10 ug/ml of lysozyme on ice for 30 minutes. Cells were sonicated at 30% duty cycle, 30% output for three 30 second bursts, and cell debris was spun down at 19,000xg for 20 minutes. Lysate was filtered and loaded onto an equilibrated 5 ml Nickel column (GE Healthcare), washed with 100 ml of buffer A, and eluted in 2 ml fractions from the column using an imidazole gradient (final concentration 200 mM in buffer A). Fractions were run on SDS-PAGE gels and visualized using Coomassie stain. Those containing ATR13 were pooled and incubated with 6His-TEV protease overnight at 4°C while dialyzing against buffer A to remove imidazole added during elution. The TEV digest was then loaded onto an equilibrated nickel column and flow through containing cleaved ATR13 was collected; other contaminants and uncleaved 6His-ATR13 remained bound to the column. The flow through was then concentrated to a volume of 500 ul resulting in a 1 mM to 3 mM protein solution using a Millipore spin column (3,000 MW) and dialyzed against 20 mM phosphate buffer pH 7.1, 150 mM NaCl.

### Making antibody and affinity purification

Four aliquots of 1 mg/ml 6His-Δ19 ATR13 Emco5 protein in 500 ul were sent to Covance Inc. (Princeton, NJ) for custom antibody production. Two New Zealand white rabbits were used in the standard 118-day protocol and bleeds were checked against purified ATR13 protein on a dot blot. Antibody was enriched by affinity purification using ATR13 conjugated to CnBr-Sepharose 4B according to manufacturer's instructions (GE Healthcare).

### NMR experiments

Protein samples were prepared for NMR experiments by dissolving lyophilized protein in buffer containing 20 mM sodium phosphate pH 7.1, 150 mM sodium chloride, and 5% D_2_O. The final protein concentration for each sample was approximately 1 mM. All spectra were recorded at 25°C on a Bruker Avance 500 MHz instrument equipped with a room temperature probe, unless stated otherwise. NMR data were processed with NMRPipe [38] and were analyzed using CARA [39]. Backbone assignments were made with standard 3D heteronuclear NMR experiments including HNCACB, CBCA(CO)NH, HNCO, HN(CA)CO, as well as a 3D ^15^N NOESY-HSQC (100 ms mixing time) [40], [41]. The latter experiment was acquired on a Bruker 800 MHz instrument equipped with a room temperature probe. Sidechain ^1^H/^13^C signals were assigned with HCCH-TOCSY, (H)CCH-TOCSY, and (H)CCH-COSY experiments and a ^1^H-^15^N TOCSY-HSQC spectrum (60 ms mixing time), and were confirmed with (H)C(CO)NH, and H(CCO)NH experiments, as well as a HCCH-COSY recorded at 800 MHz [40], [41]. Magnetization transfer in (H)C(CO)NH, H(CCO)NH and ^1^H-^15^N TOCSY–HSQC spectra was poorer than would be expected for a 12 kDa protein, indicating some dynamics or transient protein-protein interactions. Phi torsion angle restraints were derived from ^3^J_HNHA_ couplings obtained from an HNHA spectrum [42]. Stereospecific assignments for the methyl groups of 2 of 4 valine and 5 of 8 leucine residues were obtained by comparison of ^1^H-^13^C HSQC spectra of 10% and fully ^13^C-labeled samples [43]. NOEs were identified in the 3D ^1^H-^15^N NOESY-HSQC spectrum and a ^1^H-^13^C NOESY-HSQC spectrum (85 ms mixing time) recorded on a Bruker Avance II 900 MHz instrument equipped with a cryoprobe. Residual dipolar couplings were measured from IPAP spectra [44] recorded on a ^15^N –labeled sample dissolved in buffer containing 12 mg/ml of Pf1 phage (Asla Biotech Ltd, Riga, Latvia). Tensor parameters were determined from a histogram of the couplings and values based on intermediate structures [45]. The magnitude of the alignment tensor and rhombicity were set to – 11 Hz and 0.3, respectively. Qualitative backbone dynamics information was obtained from a ^1^H-^15^N heteronuclear NOE experiment [46].

### Structure generation

Initial structures were calculated with Cyana (version 2.1) [47]. Residual dipolar coupling data was included in the final rounds of refinement using CNS (version 1.3) [48]. Structures were viewed and analyzed using MOLMOL [49]. In the calculations, NOEs were classified qualitatively as strong (1.8–2.7 Å), medium, (1.8 – 3.5 Å) or weak (1.8–5.0 Å), and Phi torsion angles were constrained to −60±30 deg for ^3^J_HNHA_ values less than 6 Hz. Hydrogen bonds were identified on the basis on NOEs and slow amide proton exchange rates (protection factors greater than 100 [50]). Constraints were applied between HN and O atoms (2.8–3.3 Å) and between N and O atoms (1.8–2.3 Å). Force constants for NOEs, dihedral angles, and hydrogen bonds were set to default values. Force constants for HN residual dipolar couplings were set to 0.7 Kcal mole^−1^ Hz^−1^ to yield r.m.s.d.s equal to the uncertainties in the measurements (∼ 1 Hz). Assignments have been submitted to the BioMagResBank under accession number RCSB10216 and the 20 of 200 structures with the lowest energies have been deposited in the Protein Data Bank under accession number 2LAI.

### Site directed mutagenesis

Site-directed mutants were generated using the Quikchange Lightning Site-Directed Mutagenesis kit (Stratagene) as per the manufacturer's instructions.

### Loss/Gain of function mutagenesis screens

For loss of function mutant screen, pENTR/D-Δ41 ATR13 Emco5 was subjected to random PCR mutagenesis using M13 primers and the Diversify Mutagenesis kit (Clontech) under buffer condition 4, as described in the product manual. For gain of function mutagenesis, pENTR/D-Δ41 ATR13 Emoy2 was used as template under the same conditions. PCR product from both reactions was gel purified and recombined into the pEarleygate 202 vector [51] using LR clonase, transformed into maximum efficiency DH5α (Invitrogen), and plated out on LA with kanamycin selection 25 ug/ml. The following day, colonies were harvested, miniprepped, and transformed into electrocompetent *Agrobacterium tumefaciens* GV3101. 1,200 loss of function GV3101 colonies were resuspended in induction medium (0.1 mM MES pH5.6, 0.1 mM MgCl2, 0.1 mM Acetosyringone) to an OD between 0.3–0.7. After 3 hours at room temperature, suspensions were inoculated onto transgenic *Nicotiana benthamiana* containing RPP13Nd. Plants were scored for altered hypersensitive response at 24 h, 48 h, and 72 h post inoculation. 800 gain of function GV3101 colonies derived from the ATR13 Emoy2 allele were screened in an identical fashion as described above.

### Emco and Emoy NoLS chimeras

Emco and Emoy NoLS chimeras were generated using two-step PCR fusions. For the Emco + NoLS construct, 5′ and 3′ portions of Emco were amplified from pENTR-Δ41ATR13 Emco using the following primers: 5′ caccatggcagccgccagcgaa 3′, 5′ ctctataatcttctcgt ggatgcctttagc 3′ and 5′ gcacacgatcttcatgtctccaaatctaa 3′, 5′ ctgtctgtcaagagca 3′. The NoLS insert was amplified using pENTR-Δ41ATR13 Emoy as template with the following primers: 5′cgagaagattatagaggcatacgatcgtca 3′ and 5′catgaagatcgtgtgccatcttagatttgg 3′. Products from these three reactions were run on a high percentage agarose gel and purified by expected size using the Qiaquick Gel Extraction kit from Qiagen. The purified PCR products were pooled and used as template with the following primers: 5′ caccatggcagccgccagcgaa 3′ and 5′ ctgtctgtcaagagca 3′. Product was gel purified and cloned into pENTR via the TOPO reaction (Invitrogen). The Emco+NoLS fusion was then cloned into pEG103 using LR clonase (Invitrogen). For the Emoy –NoLS construct, 5′ and 3′ regions of ATR13 Emoy were amplified using pENTR-Δ41ATR13 Emoy with the following primer sets: 5′ caccatggcag ccgccagcgaa 3′, 5′catgacgatcgtgtgcct ttataatcttctcgtggatgcc 3′, and 5′cgagaagattataaaggcacacgatcgtcatg tctccaaa 3′, 5′ctgactggcaacggc 3′. Product from these reactions was pooled and amplified using the following primer pair: 5′caccatggcagccgccagcgaa 3′, 5′ctgactggcaacggc 3′. pEG103-ATR13Emoy –NoLS was obtained by following the procedure described above.

### Microscopy and image processing

Images were obtained using an LSM 710 Confocal from Carl Zeiss, Inc. Pictures were taken with 40x or 60x objectives using whole leaf mounts of *N. benthamiana* expressing ATR13. Images were processed with ImageJ [52].

## Supporting Information

Figure S1 **Crystals of ATR13. A.** Examples of Δ53 ATR13 Emco5 crystals grown in various conditions in sitting drop trays. **B.** ATR13 crystal grown in chip format (Fluidigm, Inc). **C.** Typical diffraction pattern associated with ATR13 crystals.
(TIF)

Figure S2 **Overlay of ATR13 structure and Ran-GTP. A.** ATR13 is shown in magenta and RAN-GTP is in blue, showing that the homology here is incidental and not significant. **B.** Rotated view of the overlay of ATR13 and RAN-GTP.
(TIF)

Figure S3 **Clustal alignment of 16 isolates of ATR13 showing amino acid percent conservation.** Arrows denote helices, blue line indicates coiled regions, and the red line denotes disordered residues.
(TIF)

Figure S4 **Alternative low-energy structures selected from the family of 20 NMR structures of ATR13 Emco5. A–D.** Various representations of the ATR13 structure and their 180° rotations. Polymorphic residues are shown in orange and conserved residues are depicted in blue.
(TIF)

Figure S5 **Random loss-of-recognition (LOR) mutagenesis of ATR13 Emco5 scored for HR in RPP13Nd transgenic N. benthamiana plants. A.** Inoculations of various mutants generated by random pcr mutagenesis showing the varied timing and intensity of HR response after 24 h and 72 h. **B.** Western blot of various clones probed with α-ATR13 and ponceau for loading. **C.** A key to inoculation and expression data, consolidating complete lack of HR (red font), wildtype protein expression (yellow boxes), and residue alterations. Mutant clones marked with an asterisk had unique mutations not present in the retention-of-function mutational database or in other clones.
(TIF)

Table S1 **Various amino acid changes in ATR13 Emco5 that disrupt RPP13 recognition.** Wildtype residue identities are listed in bold next to their amino acid position. LOR mutants generated by PCR random mutatgenesis are listed in colors corresponding to whether those changes occurred as single (red), double (orange), or triple mutations (blue). Retention of recognition (ROR) mutants are listed in black next to the loss of function mutants and possess intact RPP13Nd recognition, illustrating that amino acid position's tolerance for change.
(TIFF)
