# Supplementary figures and images for: Structural Elucidation and Functional Characterization of the Hyaloperonospora arabidopsidis Effector Protein ATR13
Source: PLoS Pathog. 2011 Dec 15;7(12):e1002428. doi: 10.1371/journal.ppat.1002428 (PMC3240608; doi:10.1371/journal.ppat.1002428)

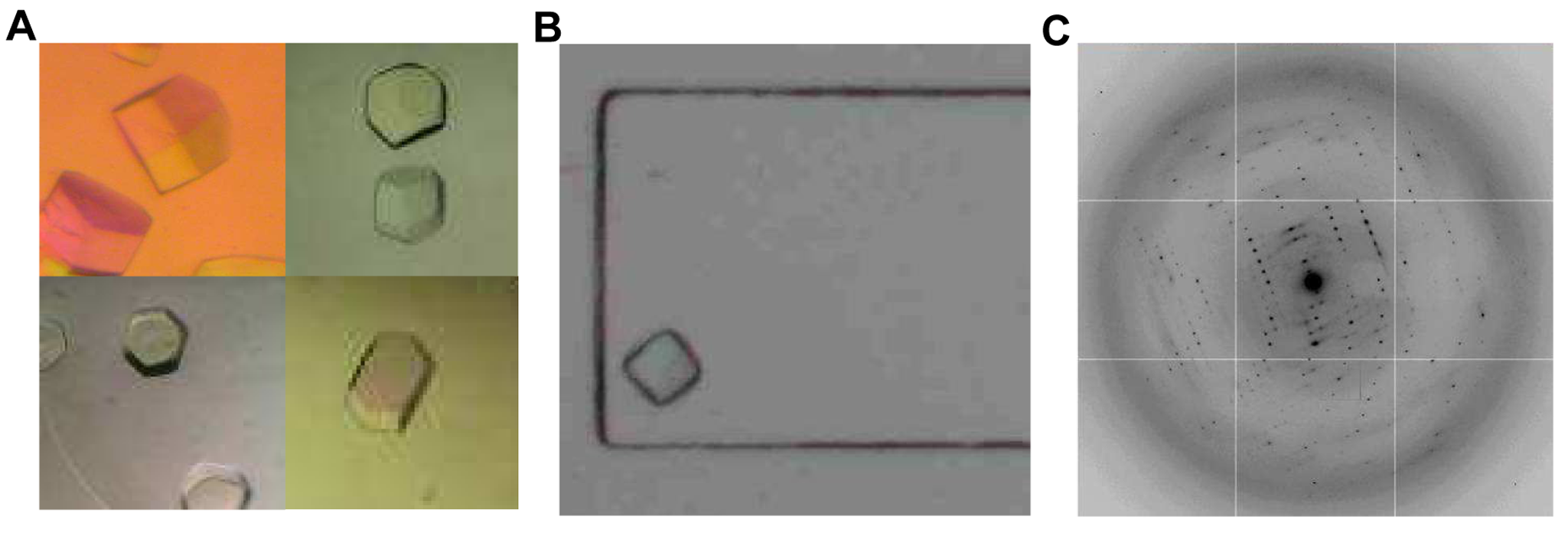

Supplement: Figure S1 — Crystals of ATR13. A. Examples of Δ53 ATR13 Emco5 crystals grown in various conditions in sitting drop trays. B. ATR13 crystal grown in chip format (Fluidigm, Inc). C. Typical diffraction pattern associated with ATR13 crystals. (TIF) [file ppat.1002428.s001.tif]

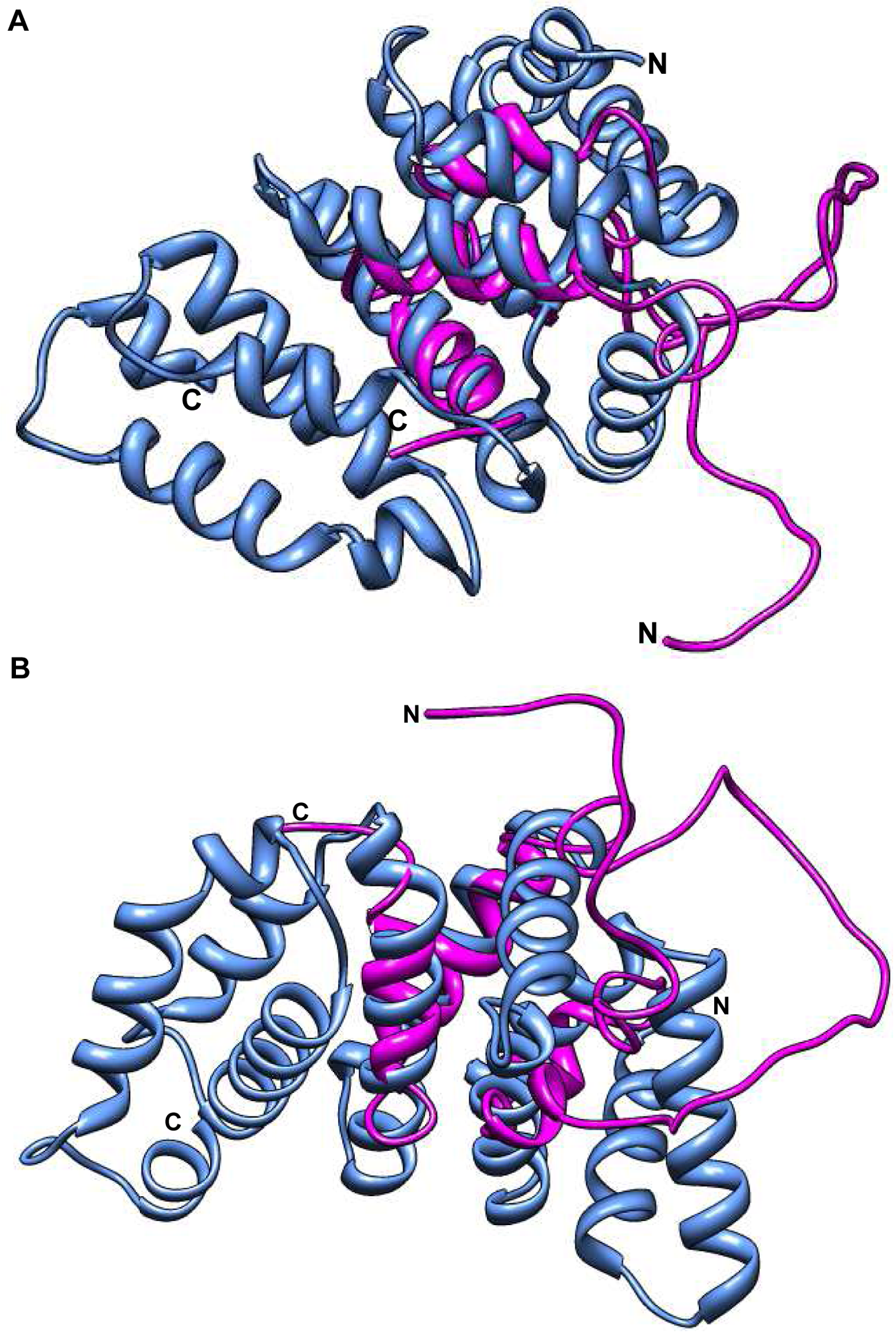

Supplement: Figure S2 — Overlay of ATR13 structure and Ran-GTP. A. ATR13 is shown in magenta and RAN-GTP is in blue, showing that the homology here is incidental and not significant. B. Rotated view of the overlay of ATR13 and RAN-GTP. (TIF) [file ppat.1002428.s002.tif]

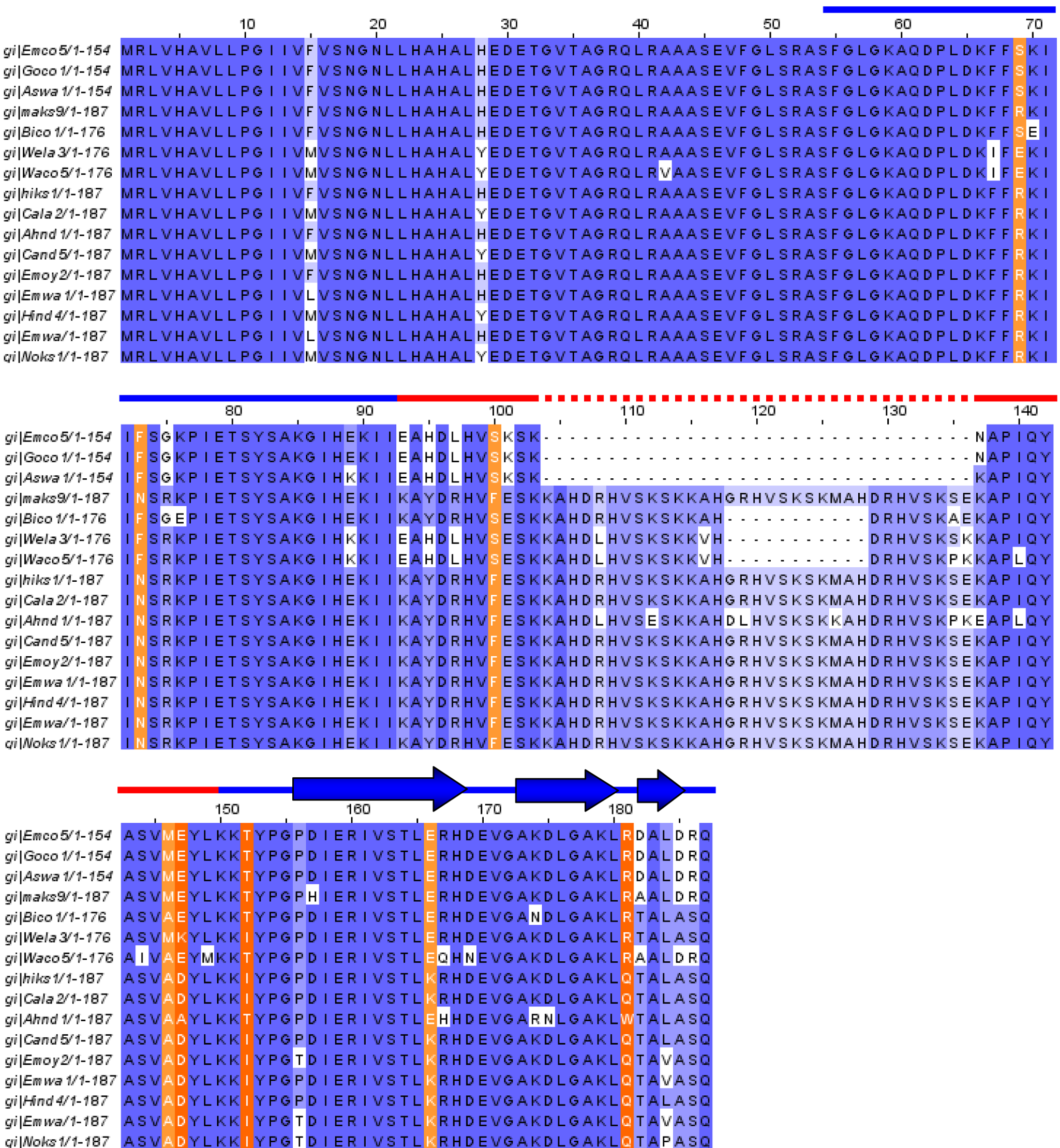

Supplement: Figure S3 — Clustal alignment of 16 isolates of ATR13 showing amino acid percent conservation. Arrows denote helices, blue line indicates coiled regions, and the red line denotes disordered residues. (TIF) [file ppat.1002428.s003.tif]

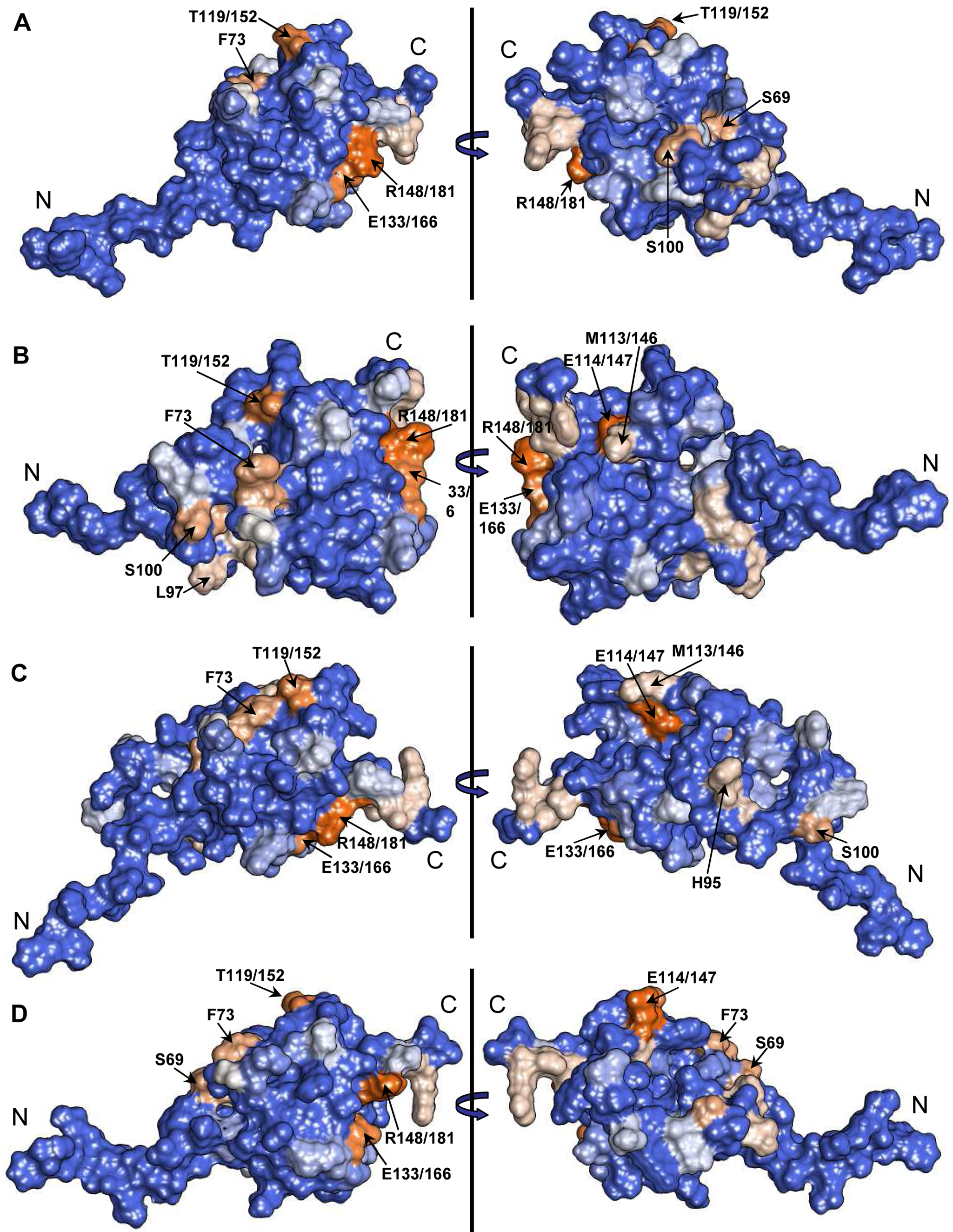

Supplement: Figure S4 — Alternative low-energy structures selected from the family of 20 NMR structures of ATR13 Emco5. A–D. Various representations of the ATR13 structure and their 180° rotations. Polymorphic residues are shown in orange and conserved residues are depicted in blue. (TIF) [file ppat.1002428.s004.tif]

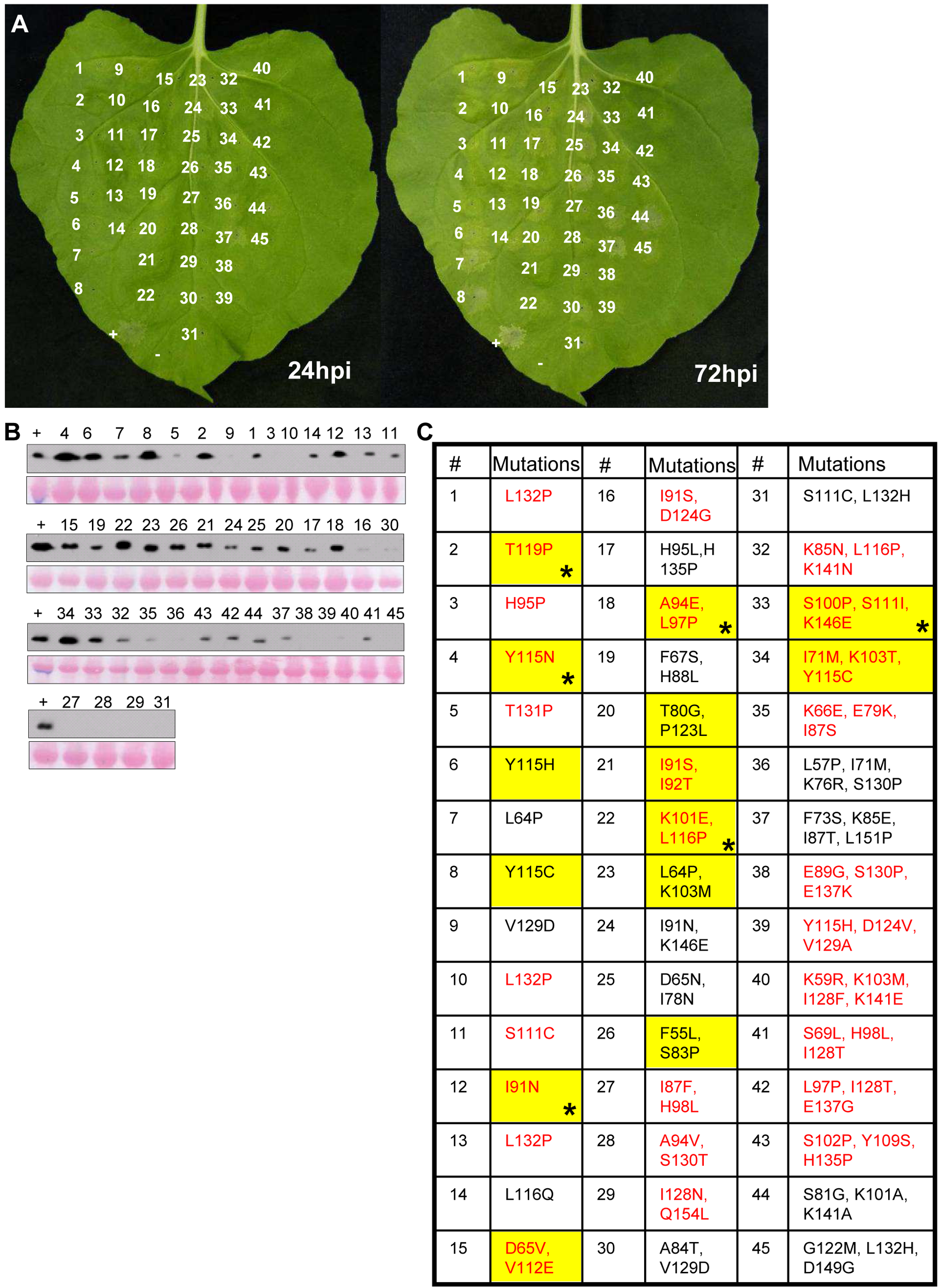

Supplement: Figure S5 — Random loss-of-recognition (LOR) mutagenesis of ATR13 Emco5 scored for HR in RPP13Nd transgenic N. benthamiana plants. A. Inoculations of various mutants generated by random pcr mutagenesis showing the varied timing and intensity of HR response after 24 h and 72 h. B. Western blot of various clones probed with α-ATR13 and ponceau for loading. C. A key to inoculation and expression data, consolidating complete lack of HR (red font), wildtype protein expression (yellow boxes), and residue alterations. Mutant clones marked with an asterisk had unique mutations not present in the retention-of-function mutational database or in other clones. (TIF) [file ppat.1002428.s005.tif]

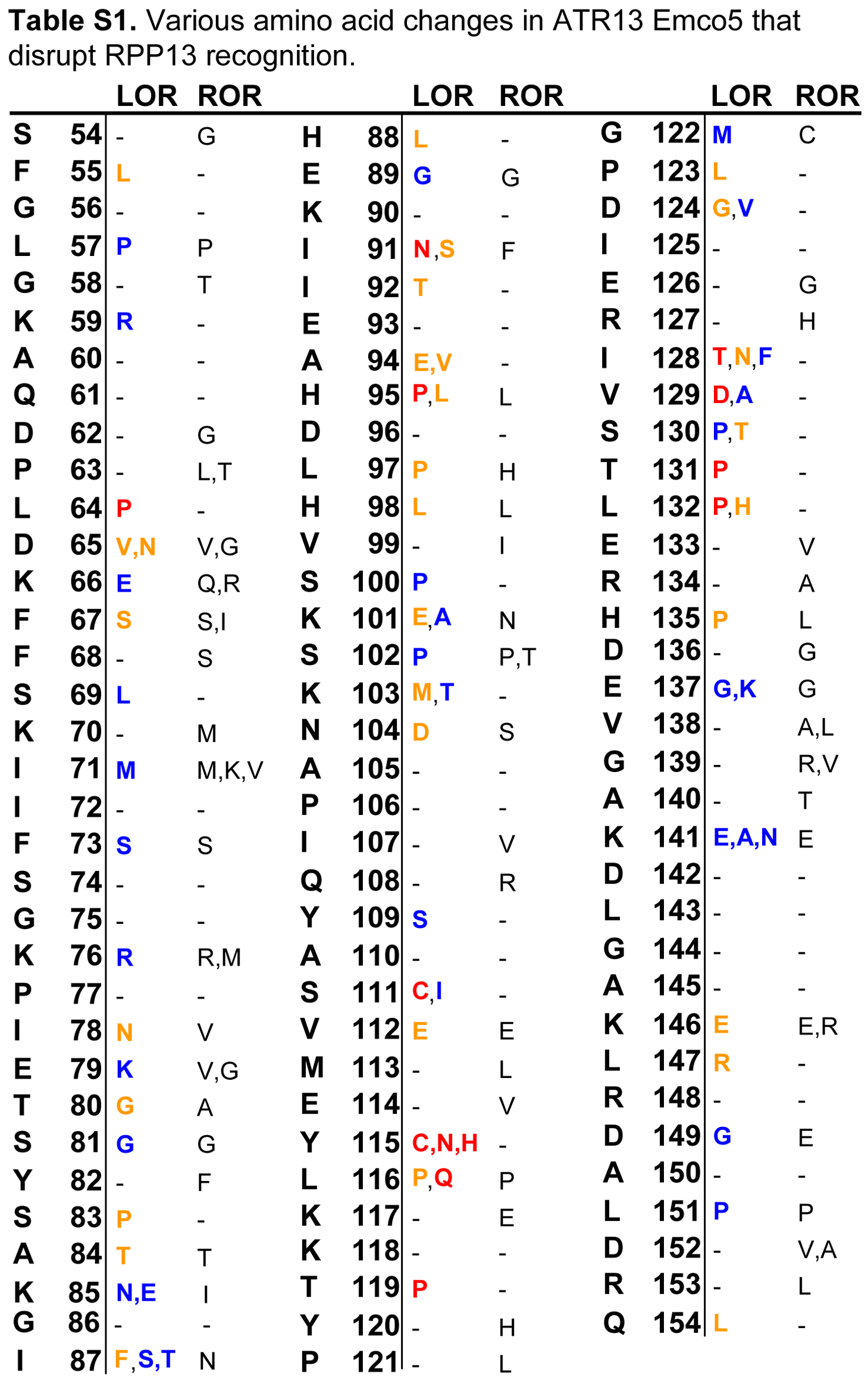

Supplement: Table S1 — Various amino acid changes in ATR13 Emco5 that disrupt RPP13 recognition. Wildtype residue identities are listed in bold next to their amino acid position. LOR mutants generated by PCR random mutatgenesis are listed in colors corresponding to whether those changes occurred as single (red), double (orange), or triple mutations (blue). Retention of recognition (ROR) mutants are listed in black next to the loss of function mutants and possess intact RPP13Nd recognition, illustrating that amino acid position's tolerance for change. (TIFF) [file ppat.1002428.s006.tiff]
